# Proteomic Profiling of Retinoblastoma-Derived Exosomes Reveals Potential Biomarkers of Vitreous Seeding

**DOI:** 10.3390/cancers12061555

**Published:** 2020-06-12

**Authors:** Angela Galardi, Marta Colletti, Chiara Lavarello, Virginia Di Paolo, Paolo Mascio, Ida Russo, Raffaele Cozza, Antonino Romanzo, Paola Valente, Rita De Vito, Luisa Pascucci, Hector Peinado, Angel M. Carcaboso, Andrea Petretto, Franco Locatelli, Angela Di Giannatale

**Affiliations:** 1 Department of Pediatric Hematology/Oncology and Cell and Gene Therapy, IRCCS, Ospedale Pediatrico Bambino Gesù, Piazza Sant’Onofrio 4, 00165 Rome, Italy; angela.galardi@opbg.net (A.G.); virginia.dipaolo@opbg.net (V.D.P.); paolo.mascio@opbg.net (P.M.); ida.russo@opbg.net (I.R.); raffaele.cozza@opbg.net (R.C.); franco.locatelli@opbg.net (F.L.); angela.digiannatale@opbg.net (A.D.G.); 2 Core Facilities-Clinical Proteomics and Metabolomics, IRCCS, Istituto Giannina Gaslini, Via Gerolamo Gaslini 5, 16147 Genoa, Italy; chiaralavarello@gmail.com (C.L.); a.petretto@gmail.com (A.P.); 3 Ophtalmology Unit, IRCCS, Ospedale Pediatrico Bambino Gesù, Piazza Sant’ Onofrio 4, 00165 Rome, Italy; antonino.romanzo@opbg.net (A.R.); paola.valente@opbg.net (P.V.); 4 Department of Pathology, IRCCS, Ospedale Pediatrico Bambino Gesù, Piazza di Sant’ Onofrio 4, 00165 Rome, Italy; rita.devito@opbg.net; 5 Department of Veterinary Medicine, University of Perugia, Via San Costanzo 4, 06126 Perugia, Italy; luisa.pascucci@unipg.it; 6 Microenvironment & Metastasis Group, Molecular Oncology Program, Spanish National Cancer Research Centre (CNIO), C/Melchor Fernández Almagro 3, 28029 Madrid, Spain; hpeinado@cnio.es; 7 Pediatric Hematology and Oncology, Hospital Sant Joan de Deu, Institut de Recerca Sant Joan de Deu, Barcelona, 08950 Esplugues de Llobregat, Spain; amontero@fsjd.org; 8 Department of Ginecology/Obstetrics & Pediatrics, Sapienza University of Rome, 00185 Roma, Italy

**Keywords:** retinoblastoma, exosomes, proteomics, biomarkers

## Abstract

Retinoblastoma (RB) is the most common tumor of the eye in early childhood. Although recent advances in conservative treatment have greatly improved the visual outcome, local tumor control remains difficult in the presence of massive vitreous seeding. Traditional biopsy has long been considered unsafe in RB, due to the risk of extraocular spread. Thus, the identification of new biomarkers is crucial to design safer diagnostic and more effective therapeutic approaches. Exosomes, membrane-derived nanovesicles that are secreted abundantly by aggressive tumor cells and that can be isolated from several biological fluids, represent an interesting alternative for the detection of tumor-associated biomarkers. In this study, we defined the protein signature of exosomes released by RB tumors (RBT) and vitreous seeding (RBVS) primary cell lines by high resolution mass spectrometry. A total of 5666 proteins were identified. Among these, 5223 and 3637 were expressed in exosomes RBT and one RBVS group, respectively. Gene enrichment analysis of exclusively and differentially expressed proteins and network analysis identified in RBVS exosomes upregulated proteins specifically related to invasion and metastasis, such as proteins involved in extracellular matrix (ECM) remodeling and interaction, resistance to anoikis and the metabolism/catabolism of glucose and amino acids.

## 1. Introduction

Retinoblastoma (RB) is the most common intraocular malignancy of childhood and accounts for 3% of all childhood cancers [1]. About 40% of cases are hereditary and usually bilateral: 10% are due to a germline mutation *of RB1* and 30% to a de novo germline mutation. The remaining 60% of cases are sporadic and non-hereditary, usually monolateral, with a somatic biallelic *RB1* inactivation arising locally within the developing retina [2,3]. In both cases, the loss of RB1 protein function, which is a tumor suppressor located on chromosome 13q14, promotes uncontrolled cell division in retinal cells determining tumor formation [4,5]. The tumor can be endophytic in the vitreous, exophytic in the subretinal space, or have a mixed presentation. Vitreous seeding may occur when the tumor penetrates the inner limiting membrane of the retina, either spontaneously or by iatrogenic mechanisms (e.g., during focal ocular treatment). When the vitreous seeding is present at diagnosis, it is defined as “primary seeding”, whereas if the disease course complicates independently of the initial growth pattern, it is termed “secondary seeding” [6]. The vitreous seeds remain a challenge in the management of intraocular RB and the enucleation of the affected eye may represent the only treatment option when the tumor is too advanced [7]. In this context, the identification of early prognostic biomarkers, which are predictive for vitreous seeding and are a reliable indicator of response to treatment, is urgently needed. Compared with other cancers, RB cannot be biopsied, due to the risk of extraocular dissemination, and much is known about the RB genetics derived from studies of tumors in enucleated eyes. Liquid biopsy is a minimally invasive alternative to surgical biopsies of solid tumors, based on the analysis of tumor-derived material in blood sample or other body fluids. Exosomes represent a novel source of biomarkers in liquid biopsies for monitoring tumor progression and drug resistance. Exosomes are cell membrane-derived nanovesicles (30–100 nM in diameter), containing RNA, microRNA, lipids and proteins. Secreted abundantly by aggressive tumor cells, those microvesicles can be isolated from several biological fluids [8]. In recent years, numerous efforts are being made to characterize the content of exosomes, both at the microRNA and protein levels [9,10]. Proteins and peptides are promising biomarkers, since they are functionally involved in biological processes. Thus, there is a correlation between their expression levels and various disease pathologies [11]. Moreover, proteomic technology platforms have developed rapidly, enhancing the precision and expedience of proteome analyses [12]. In particular, mass spectrometry has emerged as a promising approach for protein biomarker discovery, by exploring the protein content of body fluids, both in patients and controls [13].

The present study aimed to identify an exosome signature specifically associated with vitreous seeding. Here, we characterized the proteomic cargo of exosomes isolated from RB cell lines established from solid tumor tissue in the retina (retinoblastoma tumors (RBT)) and from tumor seeding in the vitreous humor (RBVS). We identified, in RBVS exosomes upregulated proteins specifically related to invasion and metastasis such as proteins involved in extracellular matrix (ECM) remodeling and interaction, resistance to anoikis and metabolism/catabolism of glucose and amino acids.

## 2. Results

### 2.1. Characterization of Exosomes Derived from Primary RB Cell Lines 

**Figure 1:**
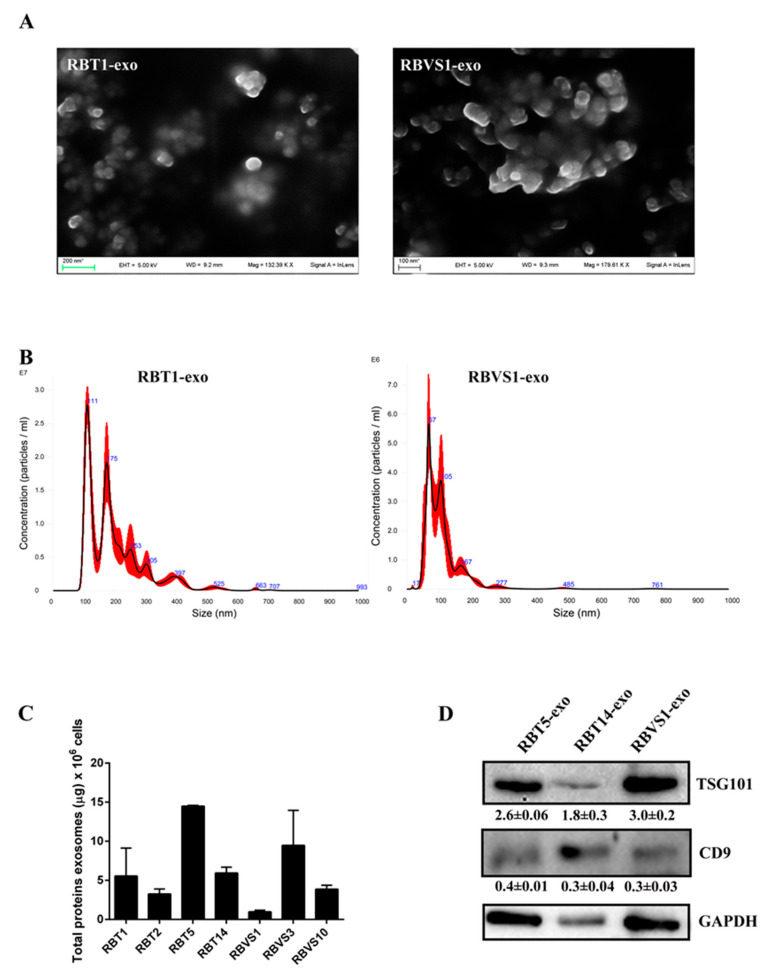
Characterization of retinoblastoma tumor (RBT)- and RB vitreous seeding (RBVS)-derived exosomes. (**A**) Scanning electron microscopy (SEM) showing a population of heterogeneous-sized exosomes isolated from representative RBT1 and RBVS1 cell lines. Scale bar: 200 nM. A higher magnification image was reported for RBVS1. Scale bar: 100 nM. (**B**) Graphics representing size distribution of nanoparticles resulting from NanoSight particle-tracking analysis of RBT1 and RBVS1, taken as representative of all RB cell lines. (**C**) Quantity of total exosomal protein for 10^6^ cells of the different RB cell lines analyzed. (**D**) Western blot analysis of the typical exosomal proteins, TSG101 and CD9 for two RBT and one RBVS representative cell lines. GAPDH was reported as loading control. Detailed information about western blot can be found at Figure S1.

Exosomes were isolated from the cell culture conditioned media of primary RB cell lines generated from primitive mass (RBT1, RBT2, RBT5, RBT14) and vitreous seeding (RBVS1, RBVS3, RBVS10) by serial ultracentrifugations, as reported in Material and Methods. Scanning electron microscopy (SEM) analysis showed single and aggregate round-shaped nano-vesicles, the majority of which ranged from 50 to 70 nM (Figure 1A). A NanoSight tracking system analysis revealed a relatively uniform size distribution of peaks from 100 to 150 nM, which is consistent with exosomes size (Figure 1B). Exosome protein concentration is reported in Figure 1C. Western blot (WB) analysis showed an enrichment of exosomal-specific proteins, such as tumor susceptibility gene 101 protein (TSG101) and the tetraspanin CD9 (Figure 1D). Altogether, these results confirmed that isolated microvesicles corresponded to exosomes.

### 2.2. Proteomic Analysis of RBT- and RBVS-Derived Exosomes

**Figure 2:**
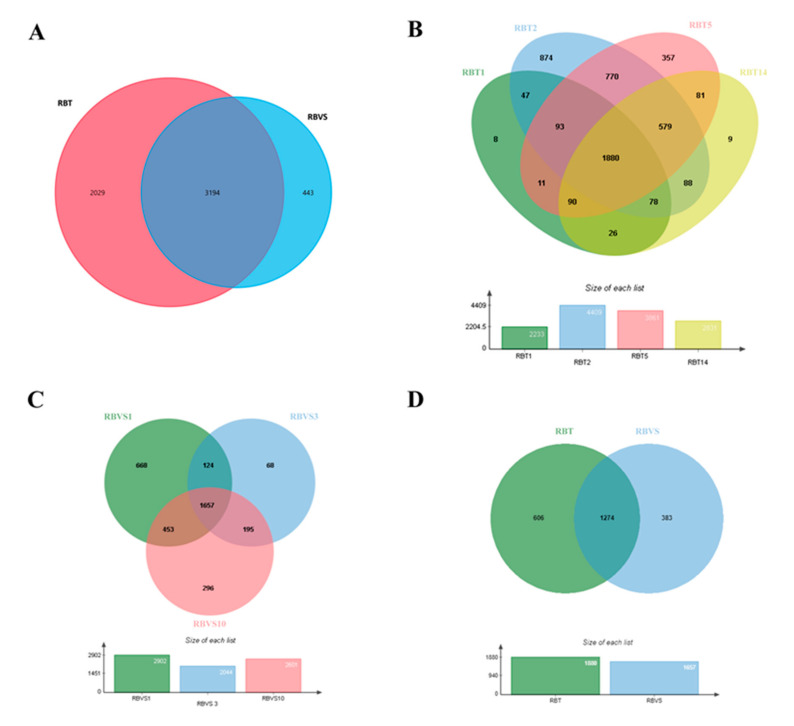
Proteomic analysis of RBT- and RBVS-derived exosomes. (**A**) Venn diagram of the total number of detected proteins in RBT and RBVS-derived exosomes. (**B**) Venn diagram of proteins identified in single RBT1, RBT2, RBT5 and RBT14 cell lines. Notably, 1880 proteins resulted present in exosomes obtained from all RBT cell lines. (**C**) Venn diagram of proteins detected in single RBVS1, RBVS3 and RBVS10 cell lines. Moreover, 1657 proteins were shared by all the samples. (**D**) Additionally, 606 proteins were exclusively present in RBT-derived exosomes, while 383 proteins were exclusively present in RBVS-derived exosomes. Furthermore, 1274 proteins were shared by the two groups.

To determine the protein profile of exosomes released by RBT and RBVS cell lines, a single shot LC-MS/MS analysis was performed. From the protein database, a total of 5666 proteins were identified in all exosomes, of which 5223 and 3637 proteins were found in RBT- and RBVS-derived exosomes, respectively. Among all proteins, 3194 proteins were shared between the two groups (Figure 2A). In particular, 2831 proteins were found in RBT14, 3861 in RBT5, 2233 in RBT1, 4409 in RBT2, 2903 in RBVS1, 2044 in RBVS3, and 2601 in RBVS10 (Figure 2B,C). The Venn diagram, depicting the extent of overlap among proteins identified in each single RBT cell line, showed 1880 proteins in common (Figure 2B). The comparison between the three RBVS cell lines highlights 1657 proteins shared (Figure 2C). Venn diagram between 1880 RBT and 1657 RBVS common proteins reveals 1274 proteins shared by the two groups, 606 and 383 proteins detected only in RBT or RBVS samples respectively (Figure 2D).

**Figure 3:**
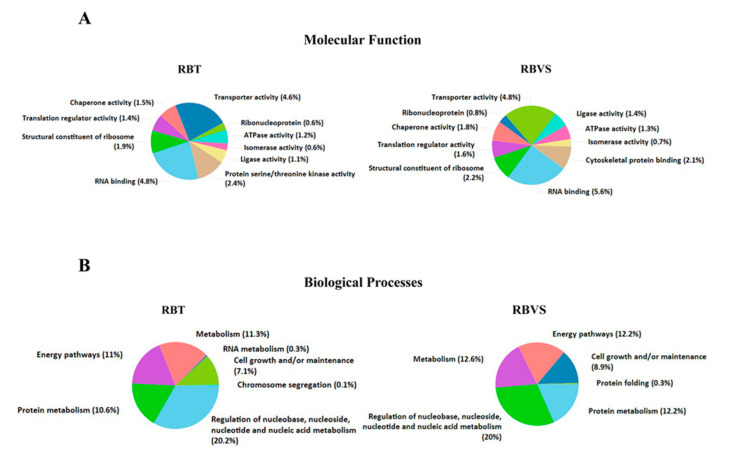
Gene enrichment of RBT- and RBVS-exosomal proteins. (**A**) Graphical representation of the molecular function and (**B**) Biological processes gene enrichment obtained by FunRich software for the 5217 and 3637 proteins, found in RBT- and RBVS-derived exosomes, respectively.

Gene enrichment analysis was performed by FunRich software [14], to characterize exosomal RBT and RBVS proteins, based on molecular function (Figure 3A) and biological processes (Figure 3B). Functionally, the proteins were associated in both groups with RNA binding (4.8% RBT; 5.6% RBVS); transporter activity (4.6% RBT; 4.8% RBVS); translation regulatory activity (1.4% RBT; 1.6% RBVS); chaperone activity (1.5% RBT; 1.8% RBVS); ligase activity (1.1% RBT; 1.4% RBVS); ATPase activity (1.2% RBT; 1.3% RBVS) and isomerase activity (0.6% RBT; 0.7% RBVS). Moreover, 1.9% and 2.2% for RBT and RBVS, respectively, clustered as structural constituent of ribosome, while 0.6% in RBT and 0.8% in RBVS as ribonucleoprotein. Proteins with serine/threonine kinase activity were present only in exosomes derived from RBT (2.4%), while cytoskeletal binding activity exclusively in exosomal RBVS associated proteins (2.1%). Clustering based on the involvement in biological processes (Figure 3B) revealed proteins involved in regulation of nucleic acid metabolism (20.2% RBT; 20% RBVS), energy pathways (11% RBT; 12.2% RBVS), metabolism (11.3% RBT; 12.6% RBVS), and cell growth and/or maintenance (7.1% RBT; 8.9% RBVS).

### 2.3. Exclusive RBT- and RBVS-Exosomal Proteins 

The comparison among all proteins highlighted 99 and 35 proteins exclusively present and statistically significant in RBT (Table 1) or RBVS-derived exosomes (Table 2), respectively. In RBT-derived exosomes were exclusively identified as the most significant, as present in all the samples analyzed, the proteins abl interactor 2 (ABI2), glutathione s-transferase Mu (GSTM), neurocan (NCAN), V-type proton ATPase subunit C 1 (ATP6V1C1), synaptosome associated protein 25 (SNAP25), serine/threonine-protein phosphatase 2A (PPP2R2A) and UBX domain protein 4 (UBXN4). In RBVS-derived exosomes were specifically found as the most significant as present in all the samples analyzed, the protein osteoglycin (OGN) and the low-density lipoprotein (LDL) receptor-related protein (LRP-1). The ABI family of adaptor proteins has been linked to signaling pathways involving the Abl tyrosine kinases and the Rac GTPase. ABI proteins localize to sites of actin polymerization in protrusive membrane structures and regulate actin dynamics in vitro, and cell morphogenesis and migration in vivo [15]. The homozygous deletion of murine *abi2* results in cell migration defects in the neocortex and hippocampus in vivo and produces abnormal phenotype in the eye. In particular, ABI2 loss leads to the incorrect orientation and migration of secondary lens fibers, probably due to defect in actin polymerization [15]. There is no clear evidence of a role of ABI2 in tumors, but previous reports suggest that ABI2 functions as a tumor suppressor since ABI2 suppresses cell growth [16] and its truncated form accelerates the tumorigenesis [17]. 

GSTM protein belongs to the superfamily of enzymes that protect normal cells, by catalyzing conjugation reactions of electrophilic compounds, including carcinogens, to glutathione [18]. In several cancers, the association between GSTM1 polymorphisms and cancer risk has been suggested through meta-analysis studies. These studies showed that the GSTM1 null phenotype increases the risk of liver, gastric, breast, and prostate cancer [19,20]. Moreover, a role for this enzyme in the cisplatin-resistance of cancer cells has been reported [21]. NCAN is expressed abundantly in developing rat retina [22], and in Müller cells correlate with the invasive phenotype in low-grade astrocytoma [23]. Müller cells are the radial glial cells of retina, spanning the entire thickness of the retina and interacting with all retinal cell types. Under pathological conditions, Müller cells are involved in retinal angiogenesis. In response to hypoxia, high glucose, and inflammation conditions, multiple signaling pathways are activated in these cells, which are involved in retinal remodeling following retinal damage [24]. Furthermore, high NCAN expression has been associated with unfavorable outcome in neuroblastoma, as it induces an undifferentiated phenotype that promotes the malignancy [25]. ATP6V1C1 belongs to the vacuolar (H+)-ATPases (or V-ATPases) family that is responsible for the acidification of intracellular compartments in eukaryotic cells [26]. It is known that V-ATPases at the cell surface play a role in maintaining an alkaline intracellular environment favorable for growth, while maintaining an acidic extracellular environment favorable for invasion [27,28]. ATP6V1C1 is overexpressed cancer such as metastatic oral squamous cell carcinoma [29], oral cancer patients [30] and breast cancer [31], and has a role in tumor progression and metastasis. Synaptosomal-associated protein-25 (SNAP-25) is a component of the trans soluble *N*-ethylmaleimide-sensitive factor (NSF) attachment protein receptors (SNAREs)-complex, a key protein that mediates several cellular processes, including synaptic vesicles fusion, transmitter release, cell growth, cytokinesis and protein transport [32]. An increase in SNAP-25 expression was reported in neuroblastoma SH-SY5Y cells during neuritogenesis [33], and is overexpressed in tumor cells of prolactinomas [34]. On the other hand, a low expression level of SNAP-25 has been found in medulloblastoma tumors associated with defects in dendrite formation and in the impairment of targeted chemotherapy [35].

PPP2R2A is a major heterotrimeric serine/threonine phosphatase, counteracting the actions of Ser/Thr kinases, such as the components of mitogen activated protein kinase (MAPK) and AKT pathways, tumor suppressor pRB and p53, and cyclin-dependent kinase 1 (CDK1) substrates, which are often defective or deregulated in cancer [36]. Finally, UBXN2A protein is a positive regulator of p53, facilitating the translocation of WT-p53 to the nucleus, where p53 regulates its target genes, particularly those involved in apoptosis. For those characteristics, UBXN2A represents an important anticancer factor [37]. 

OGN, detected exclusively in all samples of RBVS, is a matrix molecule, belonging to the small leucine-rich proteoglycan (SLRP), which functions as an important component of the extracellular matrix. This protein serves as cell scaffold and is involved in collagen fibrillogenesis and cell adhesion [38]. Decreased OGN expression has been observed in a variety of different cancers, including gastric cancer [39], colorectal adenoma [40], squamous cervical and vaginal cancer [41], invasive ductal breast carcinoma [42], and laryngeal carcinoma [43]. In colorectal cancer, OGN expression reduces cell proliferation, inhibits invasion and limits cancer progression [44]. LRP-1 modulates retinal neovascularization by regulating proteolytic activity [45]. In mouse retina, LRP-1 has a role in endothelial cell proliferation and retinal neovascularization. LRP-1 knockdown results in increased PARP-1 activity and subsequent phosphorylation of both RB protein and cyclin-dependent kinase 2 (CDK2), with cell cycle progression and angiogenesis [46]. LRP-1 function depends on the tumor cell type [47]. Indeed, if low LRP-1 expression has been associated with advanced tumor stages and poor survival in several cancers (hepatocellular carcinoma [48], lung adenocarcinoma [49], melanoma [50], and Wilms tumors [51]), high LRP-1 expression has been related to advanced tumor stages (in endometrial carcinoma [52], breast cancer [53], prostate carcinomas [54], and colon cancer [55]). 

**Figure 4:**
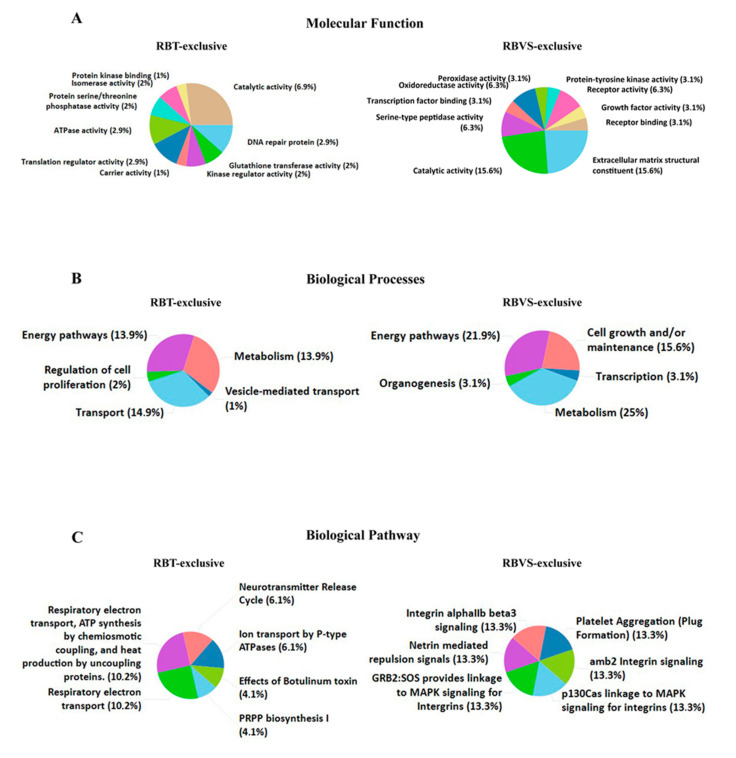
Exclusive RBT- and RBVS-exosomal proteins. Graphical representation of the molecular function (**A**), biological processes (**B**) and biological pathway (**C**) gene enrichment obtained by FunRich software for the 99 and 35 proteins found exclusively in RBT- or RBVS-derived exosomes, respectively.

We further identified molecular function (Figure 4A), biological processes (Figure 4B) and biological pathway (Figure 4C) for proteins exclusively present in RTB- or RBVS-exosomes. Among molecular function, proteins with catalytic activity were abundant in both groups (6.9% RBT; 15.6% RBVS), while extracellular matrix structural constituents were only enriched in RBVS-exosomes (15.6%). The biological processes of proteins exclusively present in RBT-derived exosomes were essentially related to the regulation of energy pathway (13.9%), metabolism (13.9%) and transport (14.9%). There was an enrichment in energy pathway (21.9%), metabolism (25%), and cell growth and/or maintenance (15.6%), for proteins exclusively present in RBVS-exosomes. Intriguingly, the analysis of biological pathway highlighted an involvement in energy-related pathways and neurotransmitter release for proteins exclusively transported by RBT-exosomes, while RBVS-exosomes were enriched with proteins involved in integrins-mediated signaling and platelet aggregation (Figure 4C). Integrins participate in cell-cell and cell-matrix interaction and have been involved in metastatic processes in several cancers [56,57,58]. In particular, αIIbβ3 integrin was first identified in platelets and later in cells derived from solid tumors, where its expression correlates with the invasive phenotype [59,60,61]. Moreover, it has been described that, integrin αIIbβ3 specifically activates c-Src to induce platelet spreading and thrombus formation [62], mediating tumor cell attachment to the vessel wall under flow condition [63]. Another interesting pathway that emerged links p130^Cas^ to integrins through the MAPK. p130^Cas^ is a multifunctional signaling adaptor protein which integrates signals generated from a variety of extracellular stimuli and regulates several cellular processes including cell death. p130^Cas^ has a particular role in anoikis, which is a programmed cell death induced upon cell detachment from extracellular matrix. Anoikis represents a critical mechanism in preventing adherent-independent cell growth and attachment to an inappropriate matrix, thus avoiding the colonizing of distant organs. p130^Cas^ is specifically cleaved during anoikis; in anoikis-sensitive cells, but not in anoikis-resistant tumor cells [64]. Another biological pathway involves Netrins, secreted or membrane bound proteins originally proposed to play an important role in development of the central nervous system and in axon guidance, but recently shown to have a pivotal role in cancer [65,66]. The expression of Netrin-1 is increased in many cancers and the binding with its receptor, uncoordinated-5 homolog (UNC5B), results in the inhibition of p53-related apoptosis [66,67].

### 2.4. Differentially Expressed Proteins in RBT- and RBVS-Derived Exosomes and Their Network 

**Figure 5:**
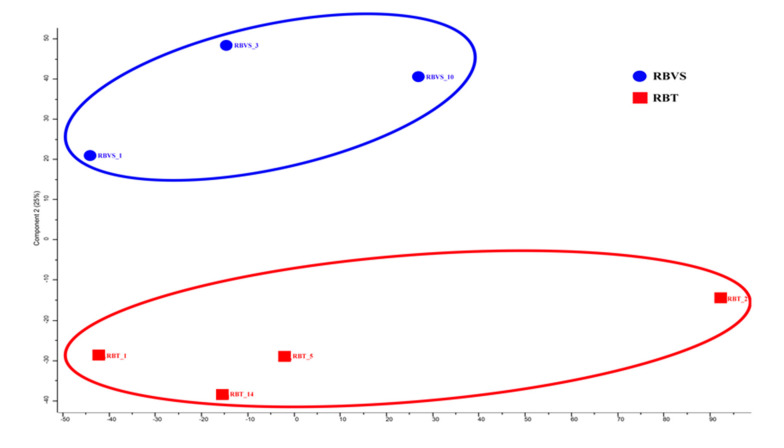
Level of similarity of individual samples. Multidimensional scaling (MDS) and k-means analysis of exosomes of RBT and RBVS proteome profile. Two-dimensional scatter plot of MDS and k-means analysis of RBT (red square) and RBVS (blue square) exosomes shows a clear discrimination between the two types of samples. The average value of the three replicates analyzed is shown.

Principal component analysis (PCA) was performed to visualize any similarities or differences between samples derived from RBT or RBVS cell lines. The two-dimensional plot shows that exosomal proteins from primary tumor (RBT1, RBT2, RBT5, RBT14) are well separated from proteins of vitreous seeding cell lines (RBVS1, RBVS3, RBVS10) (Figure 5).

**Figure 6:**
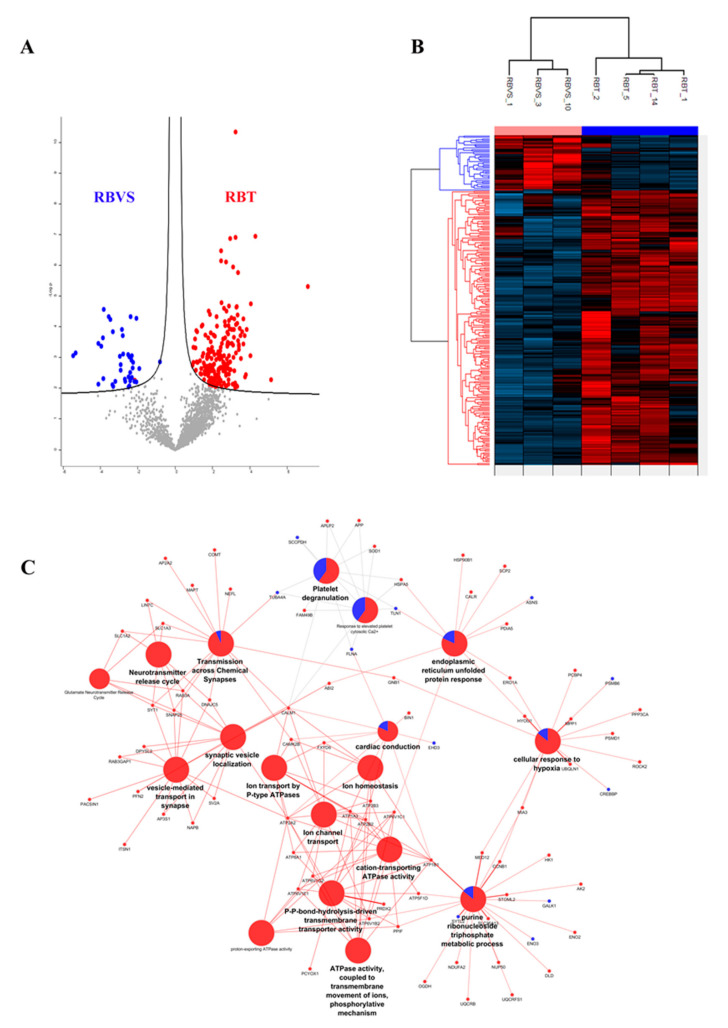
Differentially expressed proteins in RBT- and RBVS-derived exosomes and their network. (**A**) Volcano plot of differentially expressed proteins. Red dots depict proteins that display both large magnitude fold changes (*x*-axis, to the right there are proteins upregulated in RBT samples), as well as high statistical significance (−log10 of *p* value, *y*-axis). On the left side, dots representing RBVS proteins were reported, while on the right side, dots representing RBT proteins were showed. The black line shows where FDR = 0.05 and s0 = 0.1. Grey dots represent proteins for whom either fold change is <2 (log2 = 1) or *p* > 0.05. Each data point is smoothed out by a suitable Gaussian kernel; (**B**) Unsupervised hierarchical-clustered heatmap of proteins identified by *t*-test. The amount of each protein in individual samples is represented by the color scheme, in which red and blue indicate high and low proteins expression, respectively. The average of three biological replicates is shown; (**C**) Network represents the interactions between proteins that are significantly modulated between RBT and RBVS. Small nodes are proteins, while the big ones represent the functional annotation of protein clusters. The color of each node represents the fold change obtained after *t*-test: the red ones are RBT significant proteins, while the blue ones are the RBVS significant proteins. Similarly, the nodes that represent GO annotations are colored using pie charts according to the percentage of proteins either up- or down-regulated. ABI2 Abl interactor 2 AK2 Adenylate kinase 2, mitochondrial; AP23S1 AP-3 complex subunit sigma-1; AP2A AP-2 complex subunit alpha-2: APLP2 Amyloid-like protein 2; APP Amyloid beta A4 protein; ASNS Asparagine synthetase [glutamine-hydrolyzing]; ATP1A3 Sodium/potassium-transporting; ATPase subunit alpha-3; ATP1B1 Sodium/potassium-transporting ATPase subunit beta-1; ATP2A2 Sarcoplasmic/endoplasmic reticulum calcium ATPase 2; ATP2B2 Plasma membrane calcium-transporting ATPase 2; ATP2B3 Plasma membrane calcium-transporting ATPase 3; ATP5F1D ATP synthase subunit delta, mitochondrial; ATP6V1B2 V-type proton ATPase subunit B, brain isoform; ATP6V1C1 V-type proton ATPase subunit C 1; ATP6V1E1 V-type proton ATPase subunit E 1; ATP6V1G2 V-type proton ATPase subunit G 2; ATP8A1 Phospholipid-transporting ATPase IA; BIN1 Myc box-dependent-interacting protein 1; CALM1 Calmodulin-1; CALR Calreticulin; CAMK2B Calcium/calmodulin-dependent protein kinase type II subunit beta; CCNB1 G2/mitotic-specific cyclin-B1; COMT Catechol O-methyltransferase; CREBBP CREB-binding protein; DLD Dihydrolipoyl dehydrogenase, mitochondrial; DNAJ5 DnaJ homolog subfamily C member 5; DPYSL2 Dihydropyrimidinase-related protein 2; EHD3 EH domain-containing protein 3; ENO2 Gamma-enolase; Enolase; ENO3 Beta-enolase; Enolase; ERO1A ERO1-like protein alpha; FAM49B Protein FAM49B; FLNA Filamin-A; FXYD6 FXYD domain-containing ion transport regulator 6; GALK1 Galactokinase; GNB1 Guanine nucleotide-binding protein G(I)/G(S)/G(T) subunit beta-1; HK1 Hexokinase-1; HSP90B1 Endoplasmin; HSPA5 78 kDa glucose-regulated protein; HYOU1 Hypoxia up-regulated protein 1; ITSN1 Integrator complex subunit 1; LIN7C Protein lin-7 homolog C; MAPT Microtubule-associated protein tau; MED12 Mediator of RNA polymerase II transcription subunit 12; MIA3 Melanoma inhibitory activity protein 3; MPP1 55 kDa erythrocyte membrane protein; NAPB Beta-soluble NSF attachment protein; NDUFA2 NADH dehydrogenase [ubiquinone] 1 alpha subcomplex subunit 2; NEFL Neurofilament light polypeptide; NUP50 Nuclear pore complex protein Nup50; OGDH 2-oxoglutarate dehydrogenase, mitochondrial; PACSIN1 Protein kinase C and casein kinase substrate in neurons protein 1; PCBP4 Poly(rC)-binding protein 4; PCYOX1 Prenylcysteine oxidase 1; PDIA5 Protein disulfide-isomerase A5; PFN2 Profilin-2;Profilin; PPIF Peptidyl-prolyl cis-trans isomerase F, mitochondrial; PPP3CA Serine/threonine-protein phosphatase 2B catalytic subunit alpha isoform; PRDX2 Peroxiredoxin-2; PSMB6 Proteasome subunit beta type-6;Proteasome subunit beta type; PSMD1 26S proteasome non-ATPase regulatory subunit 1; RAB3A Ras-related protein Rab-3A; RAB3GAP1 Rab3 GTPase-activating protein catalytic subunit; ROCK2 Rho-associated protein kinase 2; SCCPDH Saccharopine dehydrogenase-like oxidoreductase; SCP2 Non-specific lipid-transfer protein; SLC1A2 Excitatory amino acid transporter 2;Amino acid transporter; SLC1A3 Amino acid transporter; Excitatory amino acid transporter 1; SLC25A13 Calcium-binding mitochondrial carrier protein Aralar2; SNAP25 Synaptosomal-associated protein 25; SOD1 Superoxide dismutase [Cu-Zn]; STOML2 Stomatin-like protein 2, mitochondrial; SV2A Synaptic vesicle glycoprotein 2A; SYT1 Synaptotagmin-1; SYTL2 Synaptotagmin-like protein 2; TLN1 Talin-1; TUBA4A Tubulin alpha-4A chain; UBQLN1 Ubiquilin-1; UQCRB Cytochrome b-c1 complex subunit 7; UQCRFS1 Cytochrome b-c1 complex subunit Rieske, mitochondrial.

A Student’s *t*-test analysis (FDR < 0.05 and S0 > 0.1) was performed to characterize differences and reciprocal relationships between the two groups. The significant proteins were plotted using a Volcano plot (Figure 6A) and an unsupervised hierarchical clustered HeatMap (Figure 6B). A total of 246 proteins were differentially expressed: 205 were up-regulated in RBT and 41 in RBVS-derived exosomes. We performed a Cytoscape analysis to identify molecular interaction pathways and biological functions of proteins found in RB exosomes. For this purpose, we used significant proteins obtained between the two groups through the *t*-test (RBT and RBVS-derived exosomes). In order to gain biological information from statistically modulated proteins, we have highlighted which pathways were altered in the two groups of disease, through the representation of pie charts of the enrichments in terms of biological and immunological GO annotations and using the information described in Reactome and Wiki-pathways database to simultaneously graph RBT (red) and RBVS (blue) conditions. This analysis generated a network containing 81 proteins, where the color of each node represents the fold change obtained after Student’s *t*-test: the red proteins are significantly up-regulated in RBT-derived exosomes, while the blue proteins are up-regulated in RBVS-derived exosomes (Figure 6C). Each node expresses the percentage of protein found in RBT- and RBVS-derived exosomes. The most represented pathways were involved in: (i) cellular response to hypoxia, (ii) endoplasmatic reticulum unfolded protein response, (iii) response to elevated platelet cytosolic Ca^2+^, (iv) platelet degranulation, (v) transmission across chemical synapses, (vi) neurotransmitter release vesicle localization, (vii) glutamate neurotransmitter release cycle, (viii) synaptic vesicle localization, (ix) vesicle mediated transport in synapse, (x) ion transport by P-type ATPases, (xi) cardiac conduction, (xii) ion homeostasis, (xiii) ion channel transport, (xiv) cation-transporting ATPase activity, (xv), P-P-bond-hydrolysis-driven transmembrane transport activity, (xvi) proton-exporting ATPase activity, (xvii) ATPase activity, coupled to the transmembrane movement of ions, phosphorylative mechanism, xviii) purine, ribonucleoside triphosphate metabolic process.

Exosomes derived from RBT cells showed upregulated proteins belonging to all the nodes listed above, while in exosomes isolated from RBVS cell lines, we found a significant representation of proteins involved in transmission across chemical synapses, platelet degranulation, response to elevated platelet cytosolic Ca^2+^, cardiac conduction, endoplasmatic reticulum unfolded protein response, cellular response to hypoxia, purine and ribonucleoside triphosphate metabolic process. In particular, 12 proteins were the most represented in the network for RBVS-exosomes: enolase 3 (ENO3), galactokinase-1 (GALK1), synaptotagmin like 2 (SYTL2), CREB binding protein (CREBBP), proteasome 26S subunit, non-ATPase (PSMD), Eps15 homology (EH) domain-containing protein 3 (EHD), filamin A (FLNA), talin-1 (TLN-1), asparagine synthetase (ASNS), saccharopine dehydrogenase (SCCPDH), tubulin alpha 4a (TUBA4A). Among those, ENO3, GALK1, ASNS and SCCPDH are all enzymes involved in metabolic pathways. ENO3, also known as phosphopyruvate hydratase, is a metalloenzyme responsible for the catalysis of the conversion of 2-phosphoglycerate (2-PG) to phosphoenolpyruvate (PEP), the ninth and penultimate step of glycolysis. GALK1 is an enzyme of the Leloir pathway, a metabolic pathway found in most organisms for the catabolism of β-D-galactose to glucose 1-phosphate. ASNS catalyzes the synthesis of asparagine and glutamate from aspartate and glutamine in an ATP-dependent amidotransferase reactin [68], and SCCPDH is an enzyme involved in the metabolism of the amino acid lysine, via an intermediate substance called saccharopine [69]. FLNA and TLN are important proteins for the connection of the cytoskeleton with ECM. In particular, FLNA is a well-known actin cross-linking protein that serves as a scaffold for over 90 binding partners and is involved in multiple cell functions, such as cell migration and adhesion [70]. The role of FLNA in cancer development is controversial and although its overexpression has been observed in several tumors [71,72,73,74,75,76,77,78,79,80], in some cases opposite roles have been described. For example, in breast cancer cells, the interaction of FLNA with cyclin D1 promotes migration and invasion [81], while on the other hand, the regulation of focal adhesion disassembly by FLNA leads to the suppression of breast cancer cell migration and invasion [82]. In prostate cancer, FLNA binding promoted androgen receptor (AR) localization to the nucleus [83], promoting prostate cancer progression, but once in the nucleus, it inhibited the transcriptional activity of the same transcription factor [84]. Moreover, being a scaffold protein, the function of FLNA depends on the binding partners available for its interaction.

TLN is a focal adhesion protein that links intracellular networks with the ECM, via its connection with the actin cytoskeleton and membrane integrins. TLN dysregulation can lead aberrant integrin activation and mechanotransduction, causing changes in cell spreading, migration and survival [85].

### 2.5. Comparison of Proteins Differentially Expressed between Exosomes Derived from RBT and RBVS of the Same Patient

**Figure 7:**
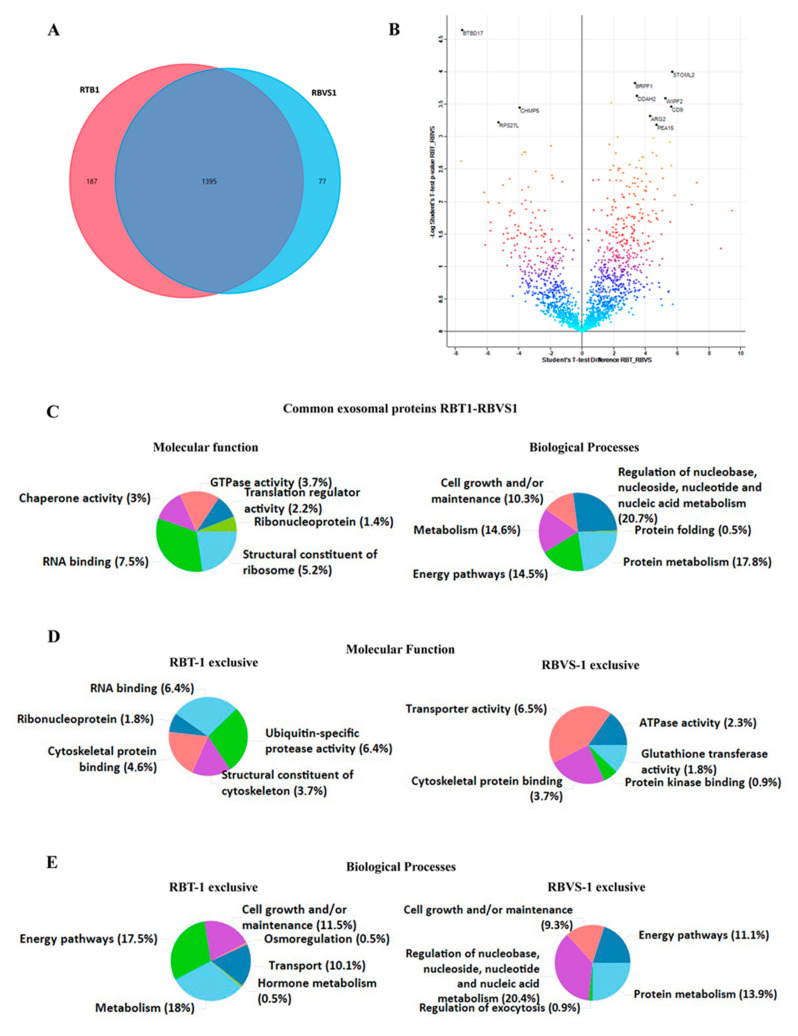
Comparison of proteins differentially expressed between exosomes, derived from RBT and RBVS of the same patient. (**A**) Venn diagram of the total number of detected proteins in RBT1 and RBVS1-derived exosomes. Notably, 187 proteins were exclusively present in RBT1-derived exosomes, while 77 proteins were exclusively present in RBVS1-derived exosomes. Overall, 1395 proteins were shared by the two cell lines. (**B**) The volcano plot illustrates the results of data obtained from the two different conditions, RBT1 and RBVS1. The proteomes are compared, starting from a value of FDR < 0.05 and S0 > 0.1. Density plot coloration highlights the similarity between the two groups, black dots are the proteins that exceed the acceptability threshold, significantly modulated. (**C**) Graphical representation of the molecular function and biological processes for common exosomal proteins of RBT1 and RBVS1 obtained with FunRich software. (**D**) Graphical representation of molecular function gene enrichment for RBT1 and RBVS1 exclusive proteins. (**E**) Graphical representation of biological processes gene enrichment for RBT1 and RBVS1 exclusive proteins.

We performed a comparison of exosomal proteins derived from RBT and RBVS cells of the same patients (RBT1 and RBVS1). This analysis showed a total of 1582 proteins for RBT1 and 1472 proteins for RBVS1, 1395 proteins were in common. Among all proteins, 187 proteins were exclusive for RBT1 and 77 for RBVS1 (Figure 7A). A total of 92 proteins were differentially expressed: 66 were up-regulated in RBT and 26 in RBVS-derived exosomes (Figure 7B). Gene enrichment analysis was performed by FunRich [14] software, to characterize common and exclusive exosomal proteins derived from RBT1 and RBVS1. Common proteins between RBT1 and RBVS1 were functionally involved in RNA binding (7.5%), were structural constituents of ribosome (5.2%), and presented a GTPase (3.7%), chaperone (3%) or translation regulator (2.2%) activity. Moreover, they were involved in biological processes such as regulation of nucleobase, nucleoside, nucleotide and nucleic acid metabolism (20.7%), protein metabolism (17.8%), energy pathways (14.5%) and cell growth and/or maintenance (10.3%) (Figure 7C). Instead proteins exclusively present in RBT1-derived exosomes were involved in molecular function such as RNA binding (6.4%) or protease activity ubiquitin-specific (6.4%), and in biological processes such as transport (10.1%), hormone metabolism (0.5%) and osmoregulation (0.5%) (Figure 7D,E). Proteins exclusively detected in RBVS1-derived exosomes were principally involved in enzymatic (5%) and transporter activity (6.5%), and in nucleic acid metabolism (20.4%) (Figure 7D,E).

## 3. Discussion 

Although intraocular RB has achieved a high cure rate through multimodal therapies, there is still an important need to develop new treatment in order to improve globe retention, in the most advanced intraocular cases, and vision preservation, as well as to avoid the late effects. Indeed, even though the management of advanced intraocular RB is improving rapidly, with enucleation progressively being supplanted by new therapeutic approaches with local delivery of standard chemotherapy [86], refractory or recurrent diffuse vitreous seeding is associated, in most cases, with the failure of eye salvage in patients with RB [7]. For these reasons, novel prognostic biomarkers, predictive for vitreous seeding dissemination and response to therapy, and new therapeutic targets are needed. Due to the risk of extraocular spread, traditional biopsy is contraindicated in RB, thus, the application of liquid biopsy as a non-invasive way represents a promising surrogate marker to perform molecular studies in RB and to define the disease more in depth. Exosomes are emerging as new circulating biomarkers in the liquid biopsy field. Cell membrane-derived nanovesicles (30–100 nM in diameter), containing RNA, microRNA, lipids and proteins, exosomes are released by several cell types and mainly by tumor cells [9,87,88]. The possibility to isolate exosomes from different sources, as well as to monitor their content in a relatively simple way, allows one to follow tumor evolution and evaluate the response to therapy over time. In this study, we characterized the proteomic cargo of exosomes isolated from RBT with those obtained from RBVS. We analyzed proteins exclusively present in all samples of RBVS and RBT-derived exosomes and proteins enriched in RBVS samples with respect to RBT. Moreover, having the primary cell lines of both primary tumor and vitreous seeding available to the same patient, we compared also these two samples. Our aim was to identify new putative biomarkers and therapeutic target associated to vitreous seeding in RB. Vitreous seeding results from a clonal selection of RB cells that are able to proliferate in the avascular vitreous environment and survive in such hypoxic conditions [89]. The ability of these cells to survive and grow under hypoxic condition depends on metabolic reprogramming and/or resistance to anoikis. The anoikis is a form of programmed cell death occurring in anchorage-dependent cells, following the detachment from the surrounding ECM. From our analysis, RBVS exosomal proteins are involved in processes of remodeling and interaction with ECM, in pathways linked to integrins signaling and in resistance to anoikis. These observations reflect the ability of RB cells that seed in the vitreous to detach from the primary retinal tumor, migrate and survive under inappropriate conditions. Alteration in integrin expression have been observed in several cancers and is thought to play a central role in cancer spread and metastasis [56]. Integrins bind to multi-adhesive ECM components, organizing the cytoskeleton and activating intracellular signaling pathways. They have been shown to affect cell shape, polarization, cytoskeletal organization, cell motility, proliferation, survival and differentiation [90]. Moreover, integrins have been identified in exosomes, and their transfer through these vesicles is implicated in the promotion of cell adhesion, migration and the determination of organotropism in metastatic dissemination [58,91]. During retinal development, ECM constituents play versatile roles, which include cellular proliferation, differentiation, migration, adhesion and maturation, as well as axonal growth and guidance [92,93,94,95]. ECM components and their remodeling have a crucial role in several diseases of the retina, such as retinal injury and cancer, where changes in the interaction of cells and ECM components occur with a consequent disruption of the retinal homeostasis [96,97]. In particular RBVS cells secrete exosomes enriched with TLN, a key cytoplasmic protein that mediates integrin adhesion to the ECM. TLN is an adaptor protein forming the core of integrin adhesion complexes by linking integrins directly to actin; TLN has been demonstrated to have an important role in cancerogenesis, promoting the invasion and anoikis’ resistance of human prostate cancer cells [98]. TLN-1 is upregulated in primary and metastatic prostate cancer compared to the normal prostate gland [98] and its downregulation led to a reduction of metastatic ability in vivo. Furthermore, the expression of TLN-1 has been reported as being significantly high in poorly differentiated prostate tumors and in cells with highly metastatic potential [99]. Intriguingly, the knockout of exosomal TLN-2 in murine lymphocytes TK1 leads to a reduced binding to the integrin ligand ICAM-1 and MAdCAM-1. Furthermore, TLN-2-deficient T-cell-exosomes were less efficiently internalized by murine bEnd.3 endothelial cells, compared with control exosomes, suggesting a critical role of TLN-2 in integrin functions in exosomes [100]. An interesting evidence is the involvement of TLN and integrin in invadosomes [101], microdomains including podosomes and invadopodia formed at the ventral surface of the cells capable of interacting with ECM and degrading it [102,103]. Invadosomes are formed by an F-actin core surrounded by a ring of regulatory and adhesive proteins such as integrins, TLN, vinculin and paxillin [101]. Furthermore, the ß3 integrin (ITGB3) founded exclusively in exosomes isolated from RBVS cells exerts several crucial roles in malignant tumor progression, such as metabolic reprogramming, maintenance of a stem-like phenotype and drug resistant acquisition [104]. Interestingly, vitreous seeds result from the clonal selection of RB cells that are able to proliferate in tumor-spheres likewise stem cells, and are prone to develop chemo-resistance properties. Additionally, GSTM1, exclusively detected in RBVS exosomes, has been implicated in cancer chemoresistance [105,106].

FLNA is another adaptor protein susceptible to proteolysis implicated in cytoplasm remodeling and interaction with the ECM. In the cytoplasm, full-length FLNA promotes the development of metastasis [107], whereas when nuclear, it was shown to be necessary for the inhibition of transcription and susceptibility to therapeutic interventions [84]. FLNA interacts with many proteins related to cancer metastasis and has a role in cancer progression [108,109,110]. Intriguingly, a role in podosomes formation and stabilization has been reported for FLNA in macrophages [111]. Through strategies of knock-out, knockdown and rescue it has been demonstrated that FLNA is involved in podosomes stability and their organization, regulates the proteolysis of the matrix and is required for podosomes formation in macrophages. A link between invadopodia formation and exosomes production has been described in the tongue squamous cell carcinoma SCC61 cell line [112]. In particular, it has been observed that the inhibition of invadopodia formation reduced exosomes secretion in culture media. On the other hand, the addition of purified exosomes or the inhibition of exosomes secretion affected the invadopodia formation, stabilization and exocytosis of proteinases indicating a key role for exosomes cargoes in the promotion of invasive activity. The fact that exosomes released by RBVS cells are enriched in proteins involved in ECM remodeling and invadosomes formation suggests their potential role in tumor dissemination during invasion and metastasis. OGN, detected exclusively in all samples of RBVS, is a small leucine rich proteoglycans of the ECM, where both structural and non-structural functions are exerted; it has been implicated in wound healing and inflammation of the eye [113]. Decreased OGN expression has been described in several malignancies, and for this reason, even if its precise role remains undefined, it is thought to be a tumor suppressor. The exclusive presence of this protein in the exosomes of RBVS cells suggests that it is eliminated by the metastatic cells to limit its tumor suppressor action or for its involvement in wound healing mechanisms related to tumor mass expansion and dissemination.

Netrins are a family of extracellular proteins that control axonal and cellular migration in embryogenesis. In particular, it has been suggested that Netrin acts through an adhesive process called haptotaxis [114], providing traction for a growth cone to navigate and generating a gradient to direct neurite growth. 

In cancer, Netrin-1 has a double role: it is a survival factor and a promoter of cell invasion [115]. In particular, netrin-1 up-regulation has been observed to be the way to gain survival advantage for several cancers, such as colorectal cancer, neuroblastoma, glioblastoma and metastatic breast cancer [116,117,118,119]. Furthermore, higher Netrin-1 expression has been correlated with invasion and metastasis in distant sites in colon carcinoma, adenoma, pancreatic ductal adenocarcinoma and breast cancer [118,120,121]. 

**Figure 8:**
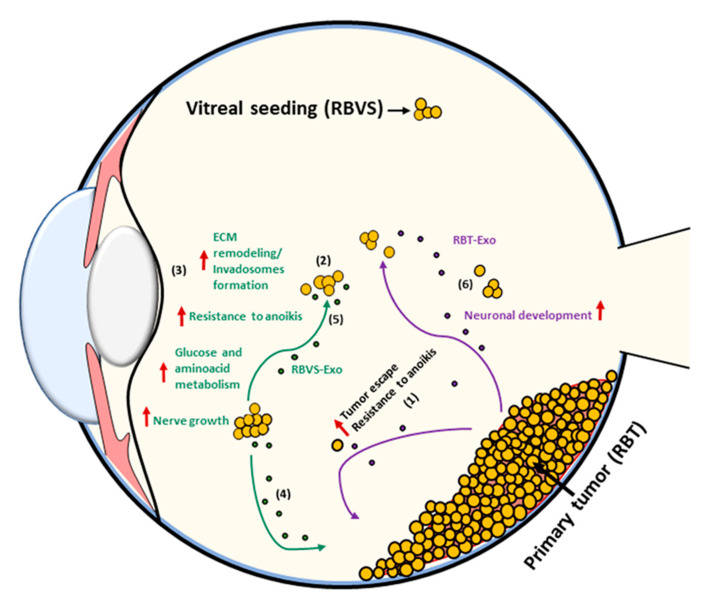
Schematic model of RBVS-exosomes role in vitreous seeding formation. From primary tumor few cells (RBT) that are able to survive in the hypoxic environment of the eye and that acquire resistance to anoikis, escape from the primary mass **(1)** and establish a metastasis in the vitreous (RBVS) **(2)**. Exosomes secreted by RBVS cells are enriched in proteins implicated in ECM remodeling/invadosomes formation, resistance to anoikis, changes in metabolism and nerve growth **(3)**. These cells can be up-taked by: (i) RBT cells inducing an invasive phenotype transferring their protein cargo and inducing anoikis resistance, formation of invadosomes, changes in metabolism in target cells **(4)** or (ii) by RBVS- cells strengthening their aggressiveness **(5)**. RBT-exosomes are enriched in proteins involved in neuronal development **(6)**.

In RBVS exosomes proteins involved in metabolic pathways or in resistance to anoikis were also found. Anoikis is a programmed cell death induced upon cell detachment from the extracellular matrix, behaving as a critical mechanism in preventing adherent-independent cell growth and attachment to an inappropriate matrix, thus avoiding the colonizing of distant organs. If non neoplastic cells undergo anoikis in response to ECM detachment, cancer cells rapidly develop several mechanisms to resist anoikis. Cancer cells can achieve resistance to anoikis through several mechanisms, such as a specific switch in integrins, which leads them to adapt to the metastatic site or change their metabolism [122]. Cells’ detachment from ECM strongly influences the metabolism of normal cells, reducing glucose uptake, glycolytic flux, mitochondrial respiration and the pentose phosphate pathway. The consequences of detachment from ECM are the reduction of both intracellular ATP and NADPH concentration, the reduction of fatty acid oxidation, the increase of ROS production and the induction of apoptosis. In recent years, it has been highlighted that the modulation of metabolic pathways in cancer cells contributes to the increase of anoikis resistance. Cancer cells metabolize high glucose levels through glycolysis, but most of the pyruvate obtained is transformed into lactate instead of being oxidized in mitochondria, a phenomenon described as the Warburg effect. Intriguingly, RBVS, which are metastatic cells able to survive in a hypoxic environment, release exosomes carrying up-regulated proteins involved in glycolysis, in glucose catabolism or in amino-acid synthesis, such as ENO3, GALK1, ASNS and SCCPDH. It is known that cells that manage to survive in the hypoxic environment of the eye and that are resistant to anoikis are able to escape from the primitive tumor site and establish a metastasis in the vitreous [89]. Based on our evidence, we hypothesize that these cells are able to produce exosomes loaded with proteins that facilitate ECM remodeling, resistance to anoikis and changes in metabolism (Figure 8). The fate and function of these exosomes depend on which cell they interact with. Indeed, exosomes released in the vitreous by vitreous seeding cells can be both incorporated by metastatic cells in an autocrine way, strengthening the aggressive phenotype, or reach sites as far away as the cells of the primary tumor. In target cells, RBVS-derived exosomes may transfer proteins involved in invadosomes formation inducing an invasive phenotype that, together with the increased resistance to anoikis and changes in metabolism, makes the cells able to escape from the primitive mass, digest the matrix and survive in a hypoxic environment in the vitreous. Moreover, Netrin-enriched exosomes secreted by vitreous seeding cells could generate a gradient that drives nerve growth and facilitates tumor innervation, which is an emerging feature of tumor progression [123]. 

## 4. Materials and Methods 

### 4.1. Cells and Cell Culture

Human RB cell lines HSJD-RBT1, HSJD-RBT2, HSJD-RBT5, HSJD-RBT14 and HSJD-RBVS1, HSJD-RBVS3, HSJD-RBVS10 were obtained from enucleated eyes of RB patients at Hospital Sant Joan de Deu (HSJD, Barcelona, Spain) and were kindly provided by Dr. Angel Montero Carcaboso. Specimens were collected from two possible sources: solid tumor tissue in the retina (RB tumor; RBT) and tumor seeding in the vitreous humor (RB vitreous seedings; RBVS) [124]. Cells were cultured as floating tumor-spheres in neural stem cell medium (serum-free), consisting of Neurobasal-A Medium (1×, ThermoFisher, Waltham, MA, USA), plus DMEM/F-12 (1×, ThermoFisher, Waltham, MA, USA) supplemented with HEPES Buffer Solution (1M, ThermoFisher, Waltham, MA, USA), Sodium pyruvate mem 100 mM (CE, ThermoFisher, Waltham, MA, USA), MEM Non-essential Amino Acids Solution 10 mM (100×, ThermoFisher, Waltham, MA, USA), GlutaMAX-I Supplement (100×, ThermoFisher, Waltham, MA, USA), Antibiotic-Antimycotic (100×, ThermoFisher, Waltham, MA, USA) B27 Supplement Minus Vitamin-A (50×, ThermoFisher, Waltham, MA, USA), Recombinant Human-basic FGF (20 ng/mL, Peprotech, Cranbury, NJ, USA), Recombinat Human EGF (20 ng/mL, Peprotech, Cranbury, NJ, USA), Human PDGF-AA (20ng/mL, Peprotech, Cranbury, NJ, USA), Human PDGF-BB (10 ng/mL, Peprotech, Cranbury, NJ, USA) and Heparin Solution 0.2% (2 µg/mL, Sigma Aldrich, St. Louis, MO, USA), as previously described [125]. Cell lines were maintained at 37 °C in a 5% (*v/v*) CO_2_ humidified incubator.

### 4.2. Exosomes Purification and Characterization

Exosomes were purified from culture media after 3–4 days culture by sequential centrifugation. Supernatants were centrifuged at 500× *g* for 10 min to remove cell contamination, then at 12,000× *g* for 20 min to eliminate possible apoptotic bodies and large cell debris. Then, exosomes were collected by spinning at 100,000× *g* for 70 min, twice, using a 70Ti rotor (Beckman Coulter, Fullerton, CA, USA). The final pellet was resuspended in PBS or specific lysis buffer for proteomic analysis. Three different biological replicates were prepared for each cell line, and analyzed both for characterization and mass spectrometry analysis. Protein concentration was measured by bicinchoninic acid assay (BCA, Pierce, Thermo Fisher Scientific, Waltham, MA, USA). Exosomes preparations were verified by SEM. Several drops of vesicles suspension (20 μL each) were placed on Parafilm. A formvar-coated nickel grid (Electron Microscopy Sciences, Hatfield, PA, USA) was placed on the top of each drop for 1 h in a humidified chamber. Grids were then fixed with 2.5% glutaraldehyde (Fluka, St. Louis, MO, USA) in phosphate buffer (PB), for 5 min at room temperature. The grids were attached on metal stubs, coated with chrome to a thickness of 10 nM and examined with a ZEISS-LEO 1525 (Laboratorio Universitario di Nanomateriali—University of Perugia). The NS500 nanoparticle characterization system (NanoSight, Salisbury, UK), equipped with a blue laser (405 nM), was used to characterize exosome size and particle number (CNIO, Madrid, Spain).

### 4.3. Western Blot 

Cell lysis buffer (Cell Lysis Buffer (10×) #9803 Cell Signalling Technology, Danvers, MA, USA), supplemented with 10 mM phenylmethylsulphonylfluoride (PMSF 93,482 Sigma, St. Louis, MO, USA), as a protease inhibitor was used to extract the proteins from exosomes. Briefly, lysates were incubated on ice for 15 min and centrifugated at 13,000× *g* for 20 min at 4 °C. Equal micrograms of proteins, quantified with BCA assay (Pierce, Thermo Scientific, Waltham, MA, USA) and boiled in SDS-loading buffer (2× Laemmli Sample Buffer BIORAD cat.#161-0737), were resolved on 10% SDS-PAGE and transferred to PVDF membranes (Immun-Blot^®^ PVDF Membrane for protein Blotting BIORAD cat.#162-0177). Blots were blocked for 1 h in TBS-T (TBS plus 0.05% Tween-20), 5% nonfat, dried milk and probed overnight at 4 °C with anti-TSG101 (4A10) ab83 (Abcam, Cambridge, UK), anti-CD9 (C-4) sc-13118 (Santa Cruz Biotechnology, Dallas, TX, USA), anti-GAPDH (D16H11) XP (Cell Signaling Technology^®^, Danvers, MA, USA). Immunocomplexes were detected with horseradish peroxidase-conjugated species-specific secondary antibodies (Santa Cruz Biotechnology, Dallas, TX, USA), followed by enhanced chemiluminescence reaction with Immobilion Western Chemiluminescence HRP substrate WBKLS0100 (Millipore, Burlington, MA, USA).

### 4.4. Sample Preparation for Mass Spectrometry, NanoLC and Mass Spectrometer Setup 

The exosome pellets were re-suspended in 50 μL of lysis buffer (6M GdmCl, 10 mM TCEP, 40 mM CAA, 100 mM Tris pH 8.5). In eppendorf cells were lysed, reduced and alkylated; lastly, 5% ProteaseMAX surfactant (Promega, Madison, WI, USA) was added to enhance protein digestion by providing a denaturing environment prior to protease addition. To the samples were added 0.3 µg LysC and 0.7 µg Trypsin in 250 µL of a dilution buffer (10% ACN, 25 mM Tris HCl pH 8.5), to dilute the ProteaseMAX to 0.1%. After overnight digestion at 37 °C, the peptides were acidified with 0.1% TFA and loaded into StageTip. [126].

The tryptic peptides, after a speed vacuum concentration, were loaded directly into a 75 μm ID × 50 cm 2 μm, 100 Å C18 column, thermostated at 55 °C, and the peptides were separated with an organic solvent at a flow rate of 250 nL/min, using a non-linear gradient of 5–45% solution B (80% CAN and 20% H_2_O, 0.1% FA) in 140 min, and analyzed using an Orbitrap Fusion Tribrid mass spectrometer (Thermo Scientific Instruments, Bremen, Germany). Orbitrap detection was used for both MS1 and MS2 measurements at resolving powers of 120 K and 30 K (at m/z 200), respectively. Data dependent MS/MS analysis was performed in top speed mode with a 2 sec cycle-time, during which precursors detected within the range of m/z 375−1500 were selected for activation in order of charge state, using CHarge Ordered Parallel Ion aNalysis (CHOPIN) [127].

Briefly, if precursor charge state is 2, then follow with CID fragmentation and by scan in the ion trap with an isolation window 1.6 m/z, AGC 3e4, maximum injection time 250 ms and normalized collision energy of 35%. If precursor charge state is 3–7 and precursor intensity is greater than 500,000, then follow with HCD fragmentation and by scan in the Orbitrap with a resolution 15,000, AgC 1e4 and maximum injection time 40 ms. If precursor charge state is 3–7 and precursor intensity is below 500,000, then follow with CID fragmentation and scan in the ion trap.

The mass spectrometry proteomics data have been deposited to the ProteomeXchange Consortium via the PRIDE [128] partner repository with the dataset identifier PXD016488”. (Reviewer account details: Username: reviewer66723@ebi.ac.uk; Password: 6oxpairt) 

### 4.5. Data Analysis and Statistical Methods

MaxQuant software was used to process the raw data, setting a false discovery rate (FDR) of 0.01 for the identification of proteins, peptides and PSM (peptide-spectrum match); moreover, a minimum length of six amino acids for peptide identification was required. Andromeda engine, incorporated into MaxQuant software, was used to search MS/MS spectra against the Uniprot human database. For protein digestion, allowing for cleavage N-terminal to proline, trypsin was chosen as enzyme. Cysteine carbamidomethylation was selected as fixed modification, whereas acetylation protein N-terminal, oxidation (M) and deamidation (N, Q) have been selected as variable modifications. A tolerance of 7 ppm was set for the mass deviation of the precursor ions, while the maximum mass deviation for MS2 events was 0.5 Da. Algorithm MaxLFQ [129] was chosen for protein quantification, with the activated option ‘match between runs’ to reduce the number of the missing proteins. All bioinformatics analyses were done with the Perseus software of the MaxQuant computational platform [130]. Protein groups were filtered to require 70% valid values in at least one experimental group. The label-free intensities were expressed as base log2, and empty values were imputed with random numbers from a normal distribution for each column, to best simulate low abundance values close to noise level. For each group, a *t*-test with permutation-based FDR of 0.05 and a s0 of 0.1 was used. The Venn diagram of identified proteins was calculated using an online tool [131]. To visualize the profile of the experiment, a hierarchical clustering of resulting proteins was performed on log2 intensities after z-score normalization of the data for each exosome line, using Euclidean distances. Lastly, the network was built on significant proteins obtained by the *t*-test between two groups (RBT and RBVS) and plot the GO Enrichment using Cytoscape 3.7 and the ClueGo app.

## 5. Conclusions

In conclusion, in this study, we isolated exosomes from RB primary tumor and vitreous seeding cell lines and characterized their content with a proteomic approach. To the best of our knowledge, this is the first evidence describing a proteomic exosome signature specifically associated with vitreous seeding in RB. This characterization may represent a starting point for future analyses that allow defining exosomal markers as promising diagnostic and potential prognostic markers in RB, as well as therapeutic targets.

## Figures and Tables

**Table 1:** Name, cellular location and function of proteins exclusively present in RBT derived exosomes.

Protein ID	Protein Name	Gene Name	Location	Function
Q9NYB9	Abl interactor 2	ABI2	Cytoplasm	Binding
E9PHN7	Glutathione S-transferase Mu 1; Glutathione S-transferase Mu 2	GSTM1; GSTM2; GSTM4	Cytoplasm	amine binding activity
O14594	Neurocan core protein	NCAN	Extracellular Region or Secreted	Binding
P21283	V-type proton ATPase subunit C 1	ATP6V1C1	Cytoplasm	active transmembrane transporter activity
P60880	Synaptosomal-associated protein 25	SNAP25	Plasma Membrane	Binding
P63151	Serine/threonine-protein phosphatase 2A 55 kDa regulatory subunit B alpha isoform; Serine/threonine-protein phosphatase 2A 55 kDa regulatory subunit B delta isoform	PPP2R2A; PPP2R2D	Cytoplasm	catalytic activity
Q92575	UBX domain-containing protein 4	UBXN4	Nucleus and Endoplasmatic Reticulum	Involved in endoplasmic reticulum-associated protein degradation
O75970-5	Multiple PDZ domain protein	MPDZ	Plasma Membrane	Binding
F8W1A4	Adenylate kinase 2, mitochondrial; Adenylate kinase 2, mitochondrial, N-terminally processed	AK2	Mitochondrial Intermembrane Space	adenyl nucleotide binding
G3V461	CREATINE Kinase B-type	CKB	Cytosol	adenyl nucleotide binding
P41567	Eukaryotic translation initiation factor 1	EIF1	Nucleus	Binding
O15144	Actin-related protein 2/3 complex subunit 2	ARPC2	Cytosol	structural constituent of cytoskeleton
Q13554-3	Calcium/calmodulin-dependent protein kinase type II subunit beta	CAMK2B	Cytoskeleton	adenyl nucleotide binding
Q15054-3	DNA polymerase delta subunit 3	POLD3	Nucleus	catalytic activity
Q70UQ0	Inhibitor of nuclear factor kappa-B kinase-interacting protein	IKBIP	Endoplasmatic Reticulum	Target of p53/TP53 with pro-apoptotic function.
Q8TDM6	Disks large homolog 5	DLG5	Plasma Membrane	binding
Q96D05-2	Uncharacterized protein C10orf35	C10orf35	Membrane	
Q9C040	Tripartite motif-containing protein 2	TRIM2	Cytoplasm	transferase
Q9H3Z4	DNA J homolog subfamily C member 5	DNAJC5	Plasma Membrane	ATP-dependent protein binding
Q9UJS0-2	Calcium-binding mitochondrial carrier protein Aralar2	SLC25A13	Mitochondrion	transport
Q9Y2Q0-3	Phospholipid-transporting ATPase IA	ATP8A1	Golgi Apparatus, Plasma Membrane, Endoplasmatic Reticulum	translocase
A0A0A0MTB8	WD repeat-containing protein 36	WDR36	Nucleus	rRNA processing
B1AM27	Protein unc-13 homolog B	UNC13B	NA	binding
A0A2R8Y6W8	TFIIH basal transcription factor complex helicase XPB subunit	ERCC3	NA	binding
A0A3B3IRW6	Glutamine and serine-rich protein 1	QSER1	NA	NA
A6PVN5	Serine/threonine-protein phosphatase 2A activator	DKFZp781M17165; PPP2R4	Cytoplasm	isomerase, rotamase
H0Y9M8	NADH dehydrogenase [ubiquinone] iron-sulfur protein 4, mitochondrial	NDUFS4	NA	catalytic activity
H3BV16	NADH dehydrogenase [ubiquinone] 1 beta subcomplex subunit 10	NDUFB10	NA	catalytic activity
K7EKE6	Lon protease homolog, mitochondrial	LONP1	Mitochondrion	adenyl nucleotide binding
M0QXF7	Myeloid-derived growth factor	MYDGF		
P27707	Deoxycytidine kinase	DCK	Nucleous	kinase transferase
P30154-4	Serine/threonine-protein phosphatase 2A 65 kDa regulatory subunit A beta isoform	PPP2R1B	Extracellular Region Or Secreted	protein phosphatase regulator activity
P35754	Glutaredoxin-1	GLRX	Cytoplasm	binding
P41236	Protein phosphatase inhibitor 2; Protein phosphatase inhibitor 2-like protein 3	PPP1R2; PPP1R2P3		protein phosphatase activity
P47985	Cytochrome b-c1 complex subunit 11; Cytochrome b-c1 complex subunit Rieske, mitochondrial; Putative cytochrome b-c1 complex subunit Rieske-like protein 1	UQCRFS1; UQCRFS1P1	Mitochondrion	translocase
Q00013-2	55 kDa erythrocyte membrane protein	MPP1	Plasma Membrane	guanylate kinase activity
Q01814-6	Plasma membrane calcium-transporting ATPase 2	ATP2B2	Plasma Membrane	active transmembrane transporter activity
Q12907	Vesicular integral-membrane protein VIP36	LMAN2	Golgi Apparatus, Endoplasmatic Reticulum	binding
Q13432	Protein unc-119 homolog A	UNC119	Cytoskeleton	binding
Q13614	Myotubularin-related protein 2	MTMR2	Endosome	catalytic activity, binding
Q15121	Astrocytic phosphoprotein PEA-15	PEA15	Cytoplasm	apoptosis, transport
Q16720-4	Plasma membrane calcium-transporting ATPase 3	ATP2B3	Plasma Membrane	active transmembrane transporter activity
Q6GMV3	Putative peptidyl-tRNA hydrolase PTRHD1	PTRHD1		hydrolase
Q6P1M0	Long-chain fatty acid transport protein 4	SLC27A4	Endoplasmatic Reticulum	binding
Q8N5M4	Tetratricopeptide repeat protein 9C	TTC9C	NA	NA
Q8NG11-2	Tetraspanin; Tetraspanin-14	TSPAN14	Plasma Membrane	enzyme binding
Q8TF74	WAS/WASL-interacting protein family member 2	WIPF2	Cytoskeleton	actin filament binding
Q92600	Cell differentiation protein RCD1 homolog	RQCD1	Nucleus	binding
Q92604	Acyl-CoA:lysophosphatidylglycerol acyltransferase 1	LPGAT1	Endoplasmatic Reticulum	catalytic activity; transferase activity
Q92784-2	Zinc finger protein DPF3	DPF3	Nucleus	binding
Q96G46	tRNA-dihydrouridine(47)synthase [NAD (P)(+)]-like	DUS3L	NA	binding
Q9BXS6-4	Nucleolar and spindle-associated protein 1	NUSAP1	Nucleus, Cytoskeleton	binding
Q9NPJ6	Mediator of RNA polymerase II transcription subunit 4	MED4	Nucleus	binding
Q9Y237	Peptidyl-prolyl cis-trans isomerase NIMA-interacting 4	PIN4	Nuclous, Mitocondrion	binding
A0A075B754	ATPase family AAA domain-containing protein 5	ATAD5	NA	ATP binding
A0A087 × 0U3	Amino acid transporter; Excitatory amino acid transporter 1	SLC1A3	Membrane	Symporter activity
A0A0A6YYL4	Coronin; Coronin-7	CORO7; CORO7-PAM16	Golgi Apparatus	NA
Q9NY35-2	Claudin domain-containing protein 1	CLDND1	Membrane	NA
A0A2R8YDI1	Homeobox protein cut-like 1; Protein CASP	CUX1	Nucleus	DNA binding
A0MZ66-8	Shootin-1	KIAA1598	Cytoskeleton	binding
A6NKD9	Coiled-coil domain-containing protein 85C	CCDC85C	Tight Junction, Adherens Junction	Developmental protein
B4DY26	Receptor protein serine/threonine kinase; TGF-beta receptor type-1	TGFBR1	Plasma Membrane	binding
B7ZAQ6-2	Golgi pH regulator A; Golgi pH regulator B	GPR89A; GPR89B	Golgi Apparatus	anion channel activity
C9JVC9	Voltage-dependent calcium channel subunit alpha-2/delta-2; Voltage-dependent calcium channel subunit alpha-2-2; Voltage-dependent calcium channel subunit delta-2	CACNA2D2	NA	binding
E7EQM8	Netrin receptor DCC	DCC	Membrane	netrin receptor activity
H0Y6H0	Lysine-specific histone demethylase 1B	KDM1B	NA	binding
O43759-2	Synaptogyrin-1	SYNGR1	Membrane	NA
O60911	Cathepsin L2	CTSV	Lysosome	aminopeptidase activity
O75182-2	Paired amphipathic helix protein Sin3b	SIN3B	Nucleus	binding
O75380	NADH dehydrogenase [ubiquinone] iron-sulfur protein 6, mitochondrial	NDUFS6	Mitochondrion	catalytic activity
O94888	UBX domain-containing protein 7	UBXN7	Nucleus	binding
O95049-5	Tight junction protein ZO-3	TJP3	Nucleus Plasma Membrane	NA
P07196	Neurofilament light polypeptide	NEFL	Cytosol, Cytoskeleton	binding
P11908	Ribose-phosphate pyrophosphokinase 2	PRPS1; PRPS2	Cytoplasm	adenyl nucleotide binding
P14635	G2/mitotic-specific cyclin-B1	CCNB1	Nucleus, Cytoskeleton	binding
P21912	Succinate dehydrogenase [ubiquinone] iron-sulfur subunit, mitochondrial	SDHB	Mitochondrion	binding
P30405	Peptidyl-prolyl cis-trans isomerase;Peptidyl-prolyl cis-trans isomerase F, mitochondrial	PPIF	Mitochondrion	binding
P56211	cAMP-regulated phosphoprotein 19	ARPP19	Cytoplasm	binding
P60468	Protein transport protein Sec61 subunit beta	SEC61B	Endoplasmic Reticulum	binding
Q13153	Non-specific serine/threonine protein kinase; Serine/threonine-protein kinase PAK 1	PAK1	Nucleus Plasma Membrane	adenyl nucleotide binding
Q13438-6	Protein OS-9	OS9	Endoplasmic Reticulum	binding
Q14118	Alpha-dystroglycan;Beta-dystroglycan; Dystroglycan	DAG1	Nucleus, Cytoskeleton, Extracellular Region, Plasma Membrane	actin binding
Q15811	Intersectin-1	ITSN1	Nucleus, Endosome, Plasma Membrane	binding
Q53EL6	Programmed cell death protein 4	PDCD4	Nucleus	nucleic acid binding
Q5T5Y3-2	Calmodulin-regulated spectrin-associated protein 1	CAMSAP1	Cytoskeleton	binding
Q68CZ6	HAUS augmin-like complex subunit 3	HAUS3	Cytoskeleton	cell division
Q86T03-2	Type 1 phosphatidylinositol 4,5-bisphosphate 4-phosphatase	TMEM55B	Plasma Membrane, Endosome, Lysosome	catalytic activity
Q8N201	Integrator complex subunit 1	INTS1	Nucleus	snRNA processing
Q8N5K1	CDGSH iron-sulfur domain-containing protein 2	CISD2	Mitochondrion, Endoplasmic Reticulum	2 iron, 2 sulfur cluster binding
Q92752	Tenascin-R	TNR	Extracellular Region or Secreted	cell adhesion
Q9BW30	Tubulin polymerization-promoting protein family member 3	TPPP3	Cytoskeleton	tubulin binding
Q9BZH6	WD repeat-containing protein 11	WDR11	Nucleus, Cytoskeleton, Golgi Apparatus	protein transport
Q9NUQ9-2	Protein FAM49B	FAM49B	Membrane	platelet degranulation, regulation of T cells
Q9NXG6	Transmembrane prolyl 4-hydroxylase	P4HTM	Endoplasmic Reticulum	binding
Q9NZB2	Constitutive coactivator of PPAR-gamma-like protein 1	FAM120A	Plasma Membrane	RNA binding
Q9UEY8-2	Gamma-adducin	ADD3	Cytoskeleton, Plasma Membrane	structural constituent of cytoskeleton
Q9UJA5	tRNA (adenine(58)–N(1))-methyltransferase non-catalytic subunit TRM6	TRMT6	Nucleus	RNA binding
Q9UKX7-2	Nuclear pore complex protein Nup50	NUP50	Nucleus	transport
Q9Y277	Voltage-dependent anion-selective channel protein 3	VDAC3	Mitochondrion	transport

**Table 2:** Name, cellular location and function of proteins exclusively present in RBVS derived exosomes.

Protein ID	Protein Name	Gene Name	Location	Function
P20774	Mimecan	**OGN**	Extracellular region or Secreted	growth factor activity
Q07954	Prolow-density lipoprotein receptor-related protein 1; Low-density lipoprotein receptor-related protein 1, 85 kDa subunit; Low-density lipoprotein receptor-related protein 1, 515 kDa subunit; Low-density lipoprotein receptor-related protein 1 intracellular domain	**LRP1**	Plasma membrane and Nucleous	Developmental Protein, Receptor
P49747	Cartilage oligomeric matrix protein	**COMP**	Extracellular region or Secreted	Binding
P05106	Integrin beta-3; Integrin beta	**ITGB3**	Plasma membrane	Cell adhesion, binding
Q5GLZ8-3	Probable E3 ubiquitin-protein ligase HERC4	**HERC4**	Cytosol	Transferase
Q8NHG7	Small VCP/p97-interacting protein	**SVIP**	Golgi apparatus, Plasma membrane, Endoplasmic reticulum	ATPase binding
Q92626	Peroxidasin homolog	**PXDN**	Extracellular region or Secreted	Oxidoreductase, Peroxidase
P23352	Anosmin-1	**KAL1**	Extracellular region or Secreted	binding
Q15582	Transforming growth factor-beta-induced protein ig-h3	**TGFBI**	Extracellular region or Secreted	binding
P30043	Flavin reductase (NADPH)	**BLVRB**	Cytoplasm	Oxidoreductase
A0A0J9YWJ4	RNA polymerase II subunit A C-terminal domain phosphatase	**CTDP1**	Nucleus	RNA polymerase
P48740-2	Mannan-binding lectin serine protease 1; Mannan-binding lectin serine protease 1 heavy chain; Mannan-binding lectin serine protease 1 light chain	**MASP1**	Extracellular region or Secreted	binding
Q9Y6C2	EMILIN-1	**EMILIN1**	Extracellular region or Secreted	Structural molecule activity
P13929-3	Beta-enolase; Enolase	**ENO3**	Cytoplasm	Binding
Q93063	Exostosin-2	**EXT2**	Endoplasmic reticulum, Golgi apparatus	binding, Catalytic activity
Q15113	Procollagen C-endopeptidase enhancer 1	**PCOLCE**	Extracellular region or Secreted	binding
P04114	Apolipoprotein B-100; Apolipoprotein B-48	**APOB**	Extracellular region or Secreted	binding
Q92793	CREB-binding protein	**CREBBP**	Nucleus	Acetyltransferase activity
S4R3D5	Aldo-keto reductase family 1 member C2; Aldo-keto reductase family 1 member C1; Aldo-keto reductase family 1 member C3; Aldo-keto reductase family 1 member C4	**AKR1C3;AKR1C2;AKR1C1;AKR1C4**	NA	Oxidoreductase activity
Q05397-5	Focal adhesion kinase 1	**PTK2**	Nucleus, Cytoskeleton, Plasma membrane	Developmental protein, Kinase, Transferase, Tyrosine-protein kinase
B4DNG0	Olfactomedin-like protein 3	**OLFML3**	NA	NA
E9PGA6	Complement C1q tumor necrosis factor-related protein 3	**C1QTNF3-AMACR; C1QTNF3**	Extracellular region or Secreted	NA
Q8N8E3	Centrosomal protein of 112 kDa	**CEP112**	Cytoskeleton	Receptor localization to synapse
O43829	Zinc finger and BTB domain-containing protein 14	**ZBTB14**	Nucleus	DNA binding
O95084	Serine protease 23	**PRSS23**	Extracellular region or Secreted	Hydrolase, Protease, Serine protease
P08493	Matrix Gla protein	**MGP**	Extracellular region or Secreted	Developmental protein
Q5SVZ6	Zinc finger MYM-type protein 1	**ZMYM1**	Nucleus	binding
Q709F0-3	Acyl-CoA dehydrogenase family member 11	**ACAD11**	Peroxisome, Mitochondrion	acyl-CoA dehydrogenase activity, Binding
Q9BT92	Trichoplein keratin filament-binding protein	**TCHP**	Mitochondrion, Plasma membrane, Cytoskeleton	Apoptosis, Cilium biogenesis/degradation
Q9NRF9	DNA polymerase epsilon subunit 3	**POLE3**	Nucleus	DNA binding

## Supplementary Materials

The following are available online at https://www.mdpi.com/2072-6694/12/6/1555/s1, Figure S1: Detailed information about western blot in Figure 1D.

## Abbreviations

RB	Retinoblastoma
RBT	Retinoblastoma tumor
RBVS	Retinoblastoma vitreous seeding
ECM	Extracellular matrix
SEM	Scanning electron microscopy
WB	Western blot
TSG101	Tumor susceptibility gene 101
ABI2	Abl Interactor 2
GSTM	Glutathione s-transferase Mu
NCAM	Neurocan
ATP6V1C1	V-type proton ATPase subunit C 1
SNAP25	Synaptosome associated protein 25
PPP2R2A	Serine/threonine-protein phosphatase 2A
UBXN4	UBX domain protein 4
OGN	Osteoglycin
LDL	Low-density lipoprotein
LRP-1	LDL receptor-related protein
V-ATPases	Vacuolar (H+)-ATPases
NSF	N-ethylmaleimide-sensitive factor
SLRP	Small leucine-rich proteoglycan
CDK2	Cyclin-dependent kinase 2
MAPK	Mitogen activated protein kinase
UNC5B	Uncoordinated-5 homolog
PCA	Principal component analysis
HeatMap	Unsupervised hierarchical clustered heatmap
ENO3	Enolase 3
GALK1	Galactokinase-1
SYTL2	Synaptotagmin like 2
CREBBP	CREB binding protein
PSMD	Proteasome 26S subunit, non-ATPase
EH	Eps15 homology
EHD	EH domain-containing protein 3
FLNA	Filamin A
TLN1	Talin 1
ASNS	Asparagine synthetase
SCCPDH	Saccharopine dehydrogenase
TUBA4A	Tubulin alpha 4a
2-PG	2-phosphoglycerate
PEP	Phosphoenolpyruvate
AR	Androgen Receptor
RIAM	Rap1-GTP-interacting adaptor molecule
DLC1	Rho GTPase-activating protein 7
HSJD	Hospital sant Joan de Deu
PB	Phosphate buffer
FDR	False discovery rate
PSM	Peptide-spectrum match
