# Supplementary material for: Proteomic Profiling of Retinoblastoma-Derived Exosomes Reveals Potential Biomarkers of Vitreous Seeding
Source: Cancers (Basel). 2020 Jun 12;12(6):1555. doi: 10.3390/cancers12061555 (PMC7352325; doi:10.3390/cancers12061555)
Supplement: Supplementary file 1 [file cancers-12-01555-s001.pdf]

*Supplementary Files*

# Proteomic Profiling of Retinoblastoma-Derived Exosomes Reveals Potential Biomarkers of Vitreous Seeding

**Angela Galardi, Marta Colletti, Chiara Lavarello, Virginia Di Paolo, Paolo Mascio, Ida Russo, Raffaele Cozza, Antonino Romanzo, Paola Valente, Rita De Vito, Luisa Pascucci, Hector Peinado, Angel M. Carcaboso, Andrea Petretto, Franco Locatelli and Angela Di Giannatale**

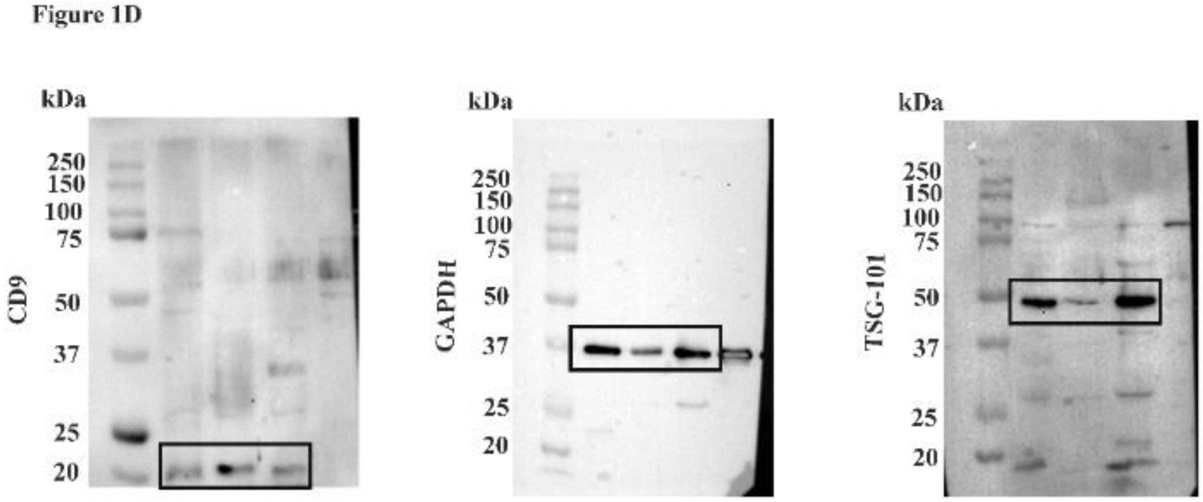


**Figure S1.** Detailed information about Figure 1D.

© 2020 by the authors. Licensee MDPI, Basel, Switzerland. This article is an open access article distributed under the terms and conditions of the Creative Commons Attribution (CC BY) license (http://creativecommons.org/licenses/by/4.0/).
